# Diagnosis of a malayan filariasis case using a shotgun diagnostic metagenomics assay

**DOI:** 10.1186/s13071-016-1363-2

**Published:** 2016-02-16

**Authors:** Dian Gao, Qiongfang Yu, Guangqiang Wang, Guitang Wang, Fan Xiong

**Affiliations:** Department of Pathogen Biology and Immunology, Medical College of Nanchang University, Nanchang, 330006 China; Department of Gastroenterology and Hepatology, Second Affiliated Hospital of Nanchang University, Nanchang, 330006 China; Qilu Hospital of Shandong University, Jinan, 250012 China; Institute of Hydrobiology, Chinese Academy of Sciences, Wuhan, 430072 China

**Keywords:** Filariasis, Parasite, Helminth, Metagenomics, *Brugia malayi*

## Abstract

**Background:**

Malayan filariasis is a lymphatic filariasis caused by *Brugia malayi*. It is easily misdiagnosed in non-endemic areas for atypical symptoms and rare diagnostic experience. A 34-year-old Chinese woman in New York presented with diffuse erythema on her body, swelling of her body, and watery, itchy, red, sore, swollen and stinging of the eyes, and severe night-time itching. No hospital that the patient visited could make a definite diagnosis by conventional diagnostic methods. It is therefore necessary to explore a new effective method to detect the pathogen that infected the patient.

**Findings:**

An unbiased metagenomic approach was used in this study. After DNA was extracted from the patient’s eye discharge sample and subcutaneous tissue sample, extended parallel sequencing was performed. The obtained raw reads were aligned to human genome to filter out the reads of the host, and the remaining reads were aligned to a candidate pathogenic protein database and four filarial genomes. The result showed that the reads of *B. malayi* accounted for an overwhelming ratio in the two samples, which indicated that the patient suffered from malayan filariasis. The subsequent therapeutic efficacy of anti-filariasis treatment validated the result of metagenomics assay.

**Conclusions:**

The present study proved that metagenomic assay can be an effective approach in the diagnosis of parasitic infection. We report a rare case of malayan filariasis from the United States.

**Electronic supplementary material:**

The online version of this article (doi:10.1186/s13071-016-1363-2) contains supplementary material, which is available to authorized users.

## Background

Lymphatic filariasis (LF) is a global health problem and is endemic in 73 countries throughout the tropics and sub-tropics, where it is a major cause of acute and chronic morbidity and a significant impediment to socioeconomic development [1]. It accounts for about 40 million chronically disabled or incapacitated people [2]. Three filarial nematodes, *Brugia malayi*, *Brugia timori*, and *Wucheria bancrofti* are responsible for lymphatic filariasis, which involves asymptomatic, acute, and chronic conditions [3].

In the past decades, the standard method to confirm a filarial infection was the identification of microfilariae in peripheral blood by microscopic examination. Besides the low sensitivities of this method, it is also inconvenient as the blood has to be collected at night, for the most prevalent filarial species (*W. bancrofti*), to coincide with the peak appearance of the microfilariae in peripheral blood. Since the 1990s, a number of techniques including detections of specific antibodies and circulating antigen and PCR array have been developed to simplify the diagnosis of filariasis and improve test reliability [4–6]. However, none of these methods have enough sensitivity and accuracy to detect all three kinds of lymphatic filariasis at the same time. Therefore a doctor easily makes misdiagnosis when patient presents with atypical symptoms, especially in non endemic areas.

Shotgun metagenomics, which is applied to the direct sequencing of DNA extracted from a sample without culture or target-specific amplification or capture, has been widely used in virus discovery [7] and bacterial pathogen detection [8]. It has rarely been used in the detection of parasitic infection except in limited related cases [9, 10]. Here we confirmed a case of malayan filariasis in a 34-year-old woman from New York, in the United States with this method.

## Case report

**Fig. 1:**
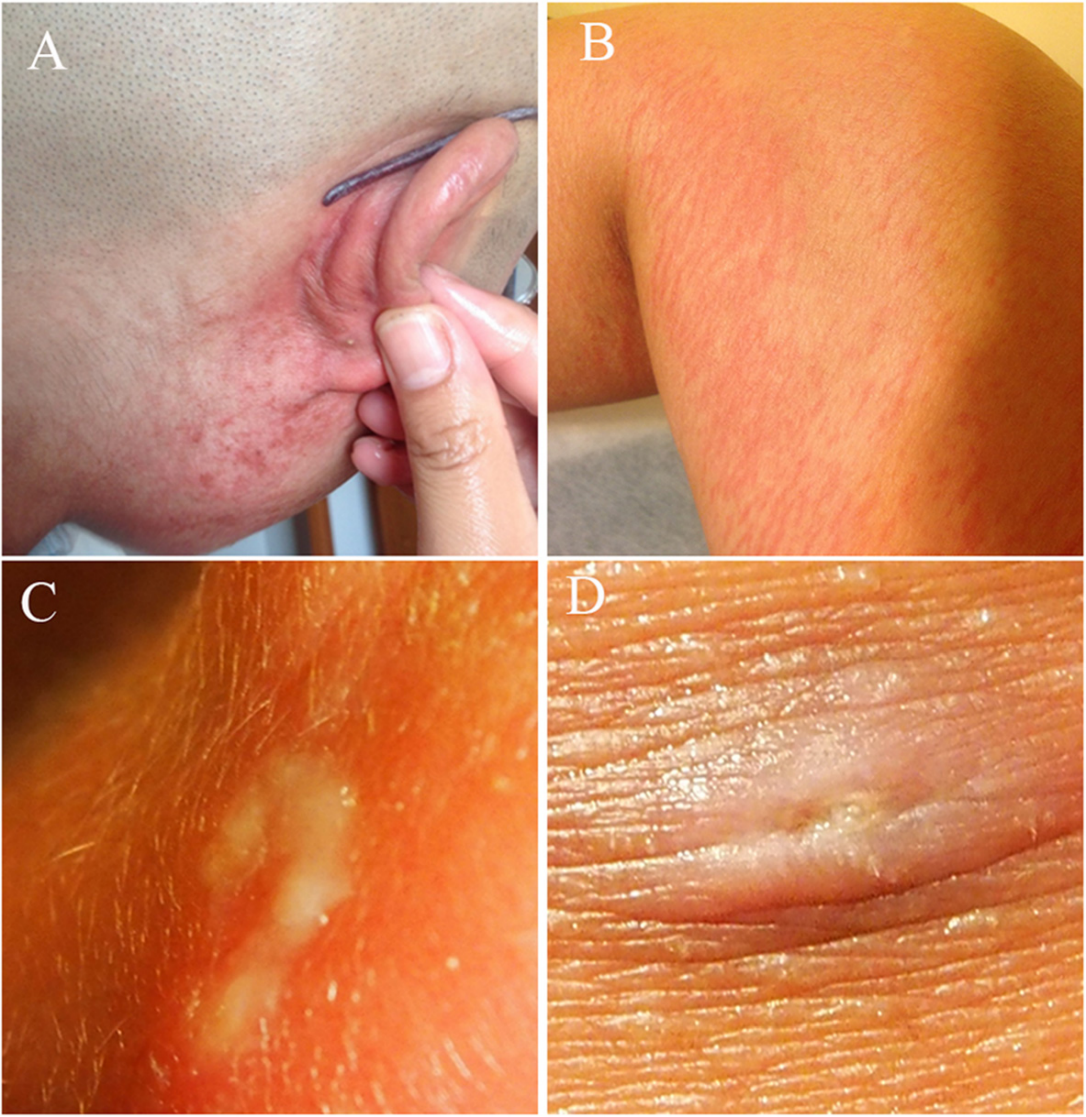
Facial and lower body clinical photographs of the patient. **a** and **b** Diffuse red rash and erythema involving the face and leg. **c** and **d** Some migratory subcutaneous nodules scattered in the retroauricular region and buttock region. Photographs courtesy of patient

A 34-year-old female Chinese student who used to study in New York presented to our services in May 2014. Over the past 12 months she had visited hospitals in the United States and China but had received no clear diagnosis and was considered to be experiencing symptoms of psychosis. She reported a one year history of diffuse erythema to her body, and watery, itchy, red, sore, swelling and stinging of the eyes, and six months of some migratory subcutaneous nodules in her scapular region, retroauricular region and buttocks region, and three months of swelling of the body (Fig. 1). She felt aggravated itching especially between 10 p.m and 3 a.m and was unable to sleep through the night. Her father showed similar but milder clinical symptoms. She was treated empirically with anti-allergy, steroids and anti-worm medication (ivermectin and mebendazole) in a local hospital in New York after presenting clinical signs. Despite these interventions, the patient got worse. Seven months ago, the patient and her father left for China. In some hospitals of China, the patient was ever suspected with helminthic infections. However, all the image and etiological examination (detection of blood-borne microfilariae), serum immunological tests (detection of IgG or/ and IgG4 or /IgM to *Spirometra mansoni Sparganum*, Cysticercus cellulose, *echinococcus granulosus*, *Clonorchis sinensis*, *Paragonimus westermani*, *Schistosoma japonicum*, *richinella spiralis*, *Angiostrongylus cantonensis*, filarial worms and *Toxoplasma gondii*) failed to confirm this suspicion. Given continued suspicion for a helminthic etiology, the patient was treated with more antiparasitic agents such as pumpkin seed, pumpkin seed oil belonging to traditional herbal medicine and praziquantel under doctor’s instruction. Initially, these drugs effectively alleviated the clinical manifestations but the effects were short lived. The patient started to have strong feelings that something was moving in the subcutaneous tissues such as finger, arm and leg joints, head, heel, and exacerbation of the itching at night.

In the following months after she returned to China, more image and pathological examinations were performed. The image examination was unremarkable. The pathological examination showed there was granulomatous inflammation in the deep dermis and subcutaneous tissues of the left little finger, and a calcific body was located in the center of granuloma (shown in Additional file 1: Figure S1). Although these examinations in general revealed a biological pathogen infection, it was still unclear as to what kind of creature this pathogen could be.

In our hospital, Qilu Hospital of Shandong University, China, an initial blood test showed leukopenia and eosinophilia with the white blood cell count 3.06 × 10^9^/L (normal range, 4–10 × 10^9^/L) with 35.70 % neutrophils (normal range 40–60 %), 35.60 % lymphocytes (normal range, 20–40 %), 16.30 % monocytes, 12.10 % eosinophils (normal range, 1 to 4 %), 0.30 % basophils (normal range, 0.5–1 %). The haemoglobin was 120 g/L (normal range, 120–150 g/L); the platelet count was 120 × 10^9^/L (normal range, 150–400 × 10^9^/L). Urinalysis and stool tests were unremarkable. The granulomas of the skin, markedly elevated proportion of eosinophilia and night-time itching indicated that the patient was possibly infected with helminthes especially filaria. However, polymerase chain reaction (PCR) testing of conservative genes of *Brugia malayi*, *Brugia timori*, and *Wucheria bancrofti* from DNA in the patient’s blood was negative. These amplified genes include cytochrome c oxidase subunit 1 (COXI) gene and ribosomal DNA 18S and ITS2. Given the absence of a diagnosis and an aggravated itching and body swelling, an unbiased metagenomic approach was ultimately used to detect the pathogen infecting the patient. The result suggested that the patient had been infected by *B. malayi*. Then the patient and her father were treated with oral diethylcarbamazine. Three months later, the symptoms of the patient disappeared.

## Methods

### Ethical approval

Written consent for clinical sample analysis was obtained from the patient. This study was approved by the Ethics Committee of Qilu Hospital of Shandong University. Because the patient’s eyes were itching terribly and often secreted foreign matter, the first sample was from the patient’s eye discharge. Another sample was collected from subcutaneous abdominal tissue which was suspected to contain pathogen. Detailed methods for nucleic acid extraction, next-generation sequencing and bioinformatics analysis are provided in Additional file 2.

## Results

**Fig. 2:**
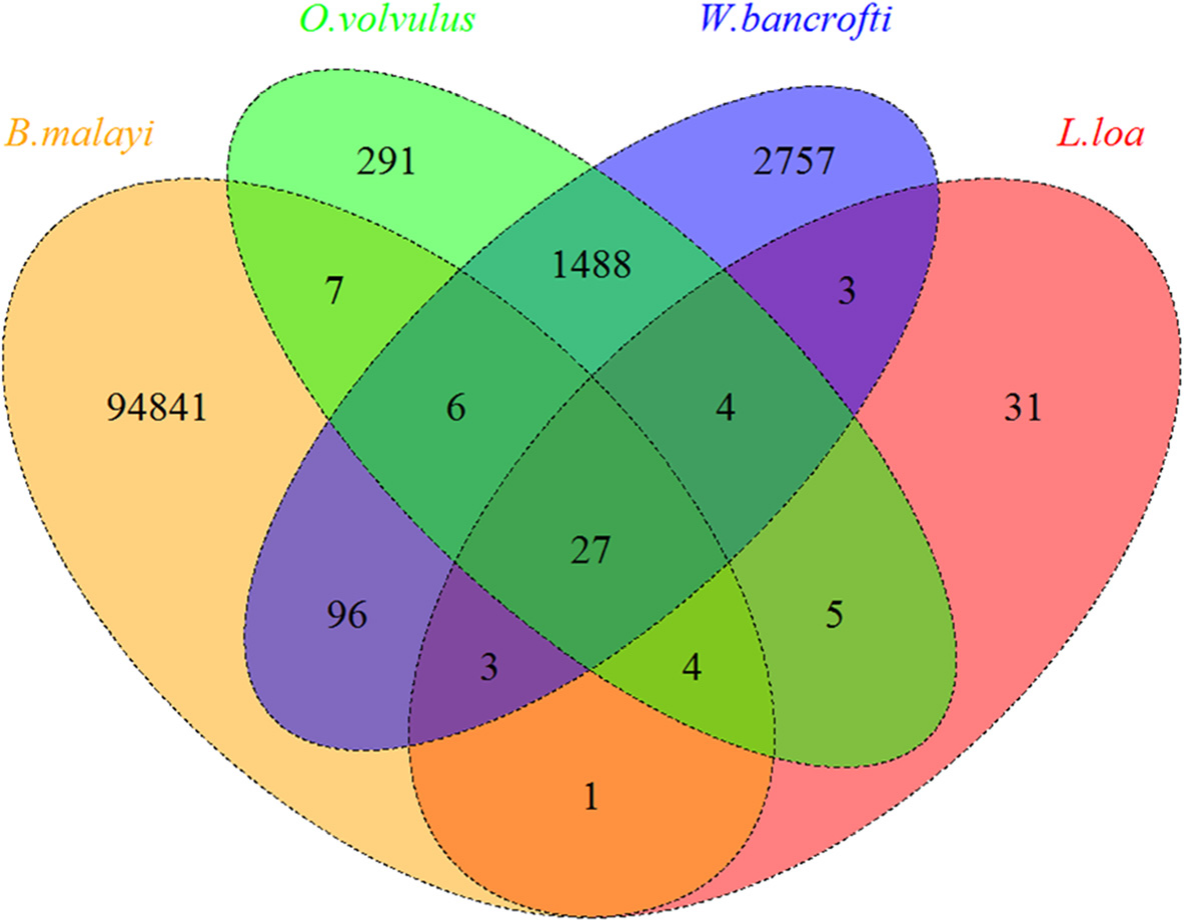
The Venn Diagram of the four sets of reads mapped to filariae of eye discharge sample

The raw reads were submitted to the DDBJ Sequence Read Archive (SRA) under the following accession numbers: PRJNA296435 and SRX1267325. As we estimated, the non-human reads accounted for a small proportion in the total sample reads (Table 1). When aligned, the non-human reads mapped to a candidate parasite protein database, the reads assigned to *B. malayi* possess the largest proportion in both samples of eye discharge (76.66 %) and subcutaneous tissue (36.45 %) (shown in Additional file 3: Figure S2 and Additional file 4: Figure S3). To find more evidence, the non-human reads from the eye discharge sample were mapped to the four available filarial genomes separately, and then overlap reads were calculated. The unique reads of *B. malayi* possess the largest ratio 96.9 % in the four filarial reads (Table 2). Although *W. bancrofti* is assigned the second-largest number of reads, it only possesses 2.8 % ratio of the total reads. The number of unique and overlapping reads among those four genomes was shown in Fig. 2. In the eye discharge sample, *B. malayi* possesses as many as 94841 unique reads. It suggests that *B. malayi* could be the most possible pathogen. Then oral diethylcarbamazine therapy was initiated for the treatment of *B. malayi*. Both the patient and her father received 1000 mg daily dose for three months. Then erythema and swelling gradually disappeared and nodules formed scars. In addition, the patient and her father did not feel itching any more.

**Table 1:** Total clean reads report and the subtraction of human reads

Sample source	Human		Non-human	
	No. of reads	Ratio	No. of reads	Ratio
Eye discharge	7955797	78.3 %	2201909	21.7 %
Subcutaneous tissue	14216801	97.8 %	318269	2.2 %

**Table 2:** The number of non-human reads mapped to each filarial genome

Sample of eye discharge	*B. malayi*	*O. volvulus*	*L. loa*	*W. bancrofti*
Number of reads mapped to filaria genomes	94985	1832	78	4384
Number of unique reads belonging to each species	94841	291	31	2757
Unique reads of each species/total filarial reads	96.9 %	0.3 %	0.03 %	2.8 %

## Discussion

The distribution of malayan filariasis is only confined to Asia. Prior to this report, malayan filariasis is rarely reported from the United States. In this case, the patient had not left New York in over a year until her father visited her. Her father reported that he had been living in Shandong Province, which was never a malayan filariasis endemic area historically [11], and he had never traveled elsewhere in the past year before he came to the United States. In addition, the patient and her father almost presented similar clinical symptoms at the same time. So it is difficult to speculate that the patient’s father had been infected in China before he came to the United States and the patient was infected through contact indirectly with her father.

The patient presented an atypical clinical manifestation compared with previous reports, where patients have some degree of lymphatic abnormality including adenolymphangitis, lymphedema, lymphadenopathy and urogenital lymphangiectasia [12]. In this case, the patient went through almost all the medical examinations in the United States and China, but almost all of which failed to draw any meaningful conclusion. Even in some hospitals, the patient was considered malayan filariasis as psychosis. So the more sensitive and broad-spectrum method of diagnostic metagenomics is promoted as a potentially life-saving gift to explore the pathogen [13]. This is the first report about detection of helminthic infections using metagenomic analysis from clinical samples, and we built an appropriate and rapid pipeline for the sequence analysis. For infection with a pathogen, metagenomic analysis should be considered when no other effective proof is presented.

## Additional files

Additional file 1: Figure S1. Histopathological findings in the nodules of the left little finger. (DOC 2022 kb) Additional file 2:
**Methods.** (DOC 31 kb) Additional file 3: Figure S2. Typing of blast hits after analyzing the eye discharge sample and detail of the phylogenetic MEGAN output. (DOC 117 kb) Additional file 4: Figure S3. Typing of blast hits after analyzing subcutaneous tissue sample and details of the phylogenetic MEGAN output. (DOC 198 kb)
