# Supplementary material for: Diagnosis of a malayan filariasis case using a shotgun diagnostic metagenomics assay
Source: Parasit Vectors. 2016 Feb 16;9:86. doi: 10.1186/s13071-016-1363-2 (PMC4754835; doi:10.1186/s13071-016-1363-2)
Supplement: Additional file 1: Figure S1. — Histopathological findings in the nodules of the left little finger. (DOC 2022 kb) [file 13071_2016_1363_MOESM1_ESM.doc]

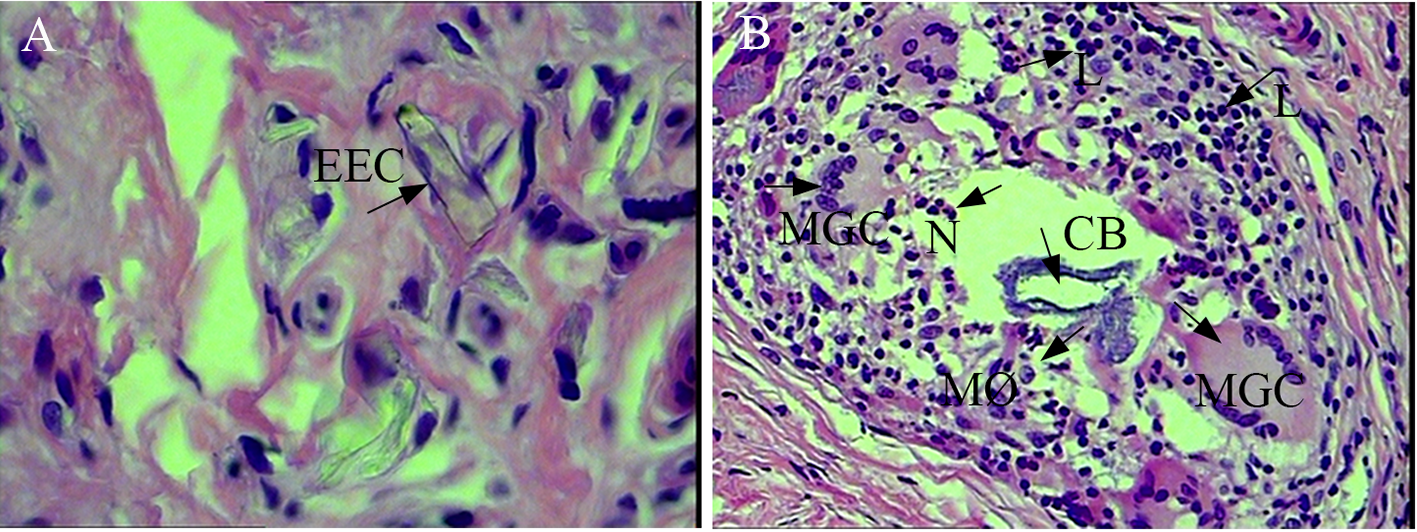


**Fig. 1S** Histopathological findings in the nodules of left little finger. **a** Exfoliated epithelial cells of pathogen in the dermis. **b** Granulomas were formed. Hematoxylin and eosin (HE) stain. EEC, exfoliated epithelial cell; L, lymphocyte; mø, macrophage; N, neutrophil; MGC, multinucleated giant cell; CB, calcific body. The representative photographs are shown at 100 × magnification.
