# Supplementary material for: Diagnosis of a malayan filariasis case using a shotgun diagnostic metagenomics assay
Source: Parasit Vectors. 2016 Feb 16;9:86. doi: 10.1186/s13071-016-1363-2 (PMC4754835; doi:10.1186/s13071-016-1363-2)
Supplement: Additional file 2: — Methods. (DOC 31 kb) [file 13071_2016_1363_MOESM2_ESM.doc]

**Methods**

**Clinical specimens collection**

Two samples were collected. The patient's eyes were itching terribly, and often secreted foreign matter. Therefore the first sample was from the patient's eye discharge. Another sample was collected from subcutaneous abdominal tissue which was suspected to contain pathogen.

**Shotgun metagenomic sequencing and analysis**

The DNA extracts from the above two samples were sheared and then bar-coded libraries were prepared. Massively parallel sequencing was performed with an Illumina Hiseq 2500 sequencer in the 101-bases paired-end mode. Initial image analysis and base calling were performed with the Illumina GAPipeline program (version 1.0) using standard parameters. To analyze sequences, In-house scripts were applied according to a previous report.^[1]^

**Taxonomic assignment**

The obtained raw reads were aligned to human genome (Homo_sapiens GRCH38) with bowtie 2-2.2.3 (http://bowtie-bio.sourceforge.net/bowtie2/index.shtml). Those reads which were not mapped to the reference sequences were extracted out as the residual sequences. Due to the low composition ratio of pathogenic sequence in the entire sequences, a screening pipeline of candidate pathogen was performed to avoid false negative caused by sequence similarity among species. A candidate pathogenic protein database was built according to all protein sequences of parasites including nematodes, cestodes and trematodes from NCBI website (National Center for Biotechnology Information). The program PAUDA (version 1.0.1, http://ab.inf.uni-tuebingen.de/software/pauda/) were used to assign the related reads of candidate pathogen by aligning the residual sequences against the candidate pathogenic protein database. The results were then parsed with the Megan software (http://ab.inf.uni-tuebingen.de/software/megan5/).

To compare to the closest species, four filarial genomes (*Brugia malayi*, [*Onchocerca volvulus*](http://www.sanger.ac.uk/resources/downloads/helminths/onchocerca-volvulus.html), *Loa loa*, and *Wuchereria bancrofti,)* were downloaded from Ensembl and Broadinstitute website (http://metazoa.ensembl.org/Brugia_malayi/Info/Index, http://metazoa.ensembl.org/Onchocerca_volvulus/Info/Index, http://metazoa.ensembl.org/Loa_loa/Info/Index, http://www.broadinstitute.org/annotation/genome/filarial_worms/MultiDownloads.html). Then the non-human reads were aligned to the downloaded genome by bowtie2 (version 2.2.3) separately. The numbers of the four sets of reads group and overlapping reads were calculated. The VennDigram package in the R software were applied to clarify the relationship among the four sets of reads group.

References

1. Taboada B, Espinoza MA, Isa P, Aponte FE, Arias-Ortiz MA, Monge-Martínez J et al. Is There Still Room for Novel Viral Pathogens in Pediatric Respiratory Tract Infections? PLoS One. 2014;9(11):e113570. doi:10.1371/journal.pone.0113570.
