# Supplementary material for: Diagnosis of a malayan filariasis case using a shotgun diagnostic metagenomics assay
Source: Parasit Vectors. 2016 Feb 16;9:86. doi: 10.1186/s13071-016-1363-2 (PMC4754835; doi:10.1186/s13071-016-1363-2)
Supplement: Additional file 3: Figure S2. — Typing of blast hits after analyzing the eye discharge sample and detail of the phylogenetic MEGAN output. (DOC 117 kb) [file 13071_2016_1363_MOESM3_ESM.doc]

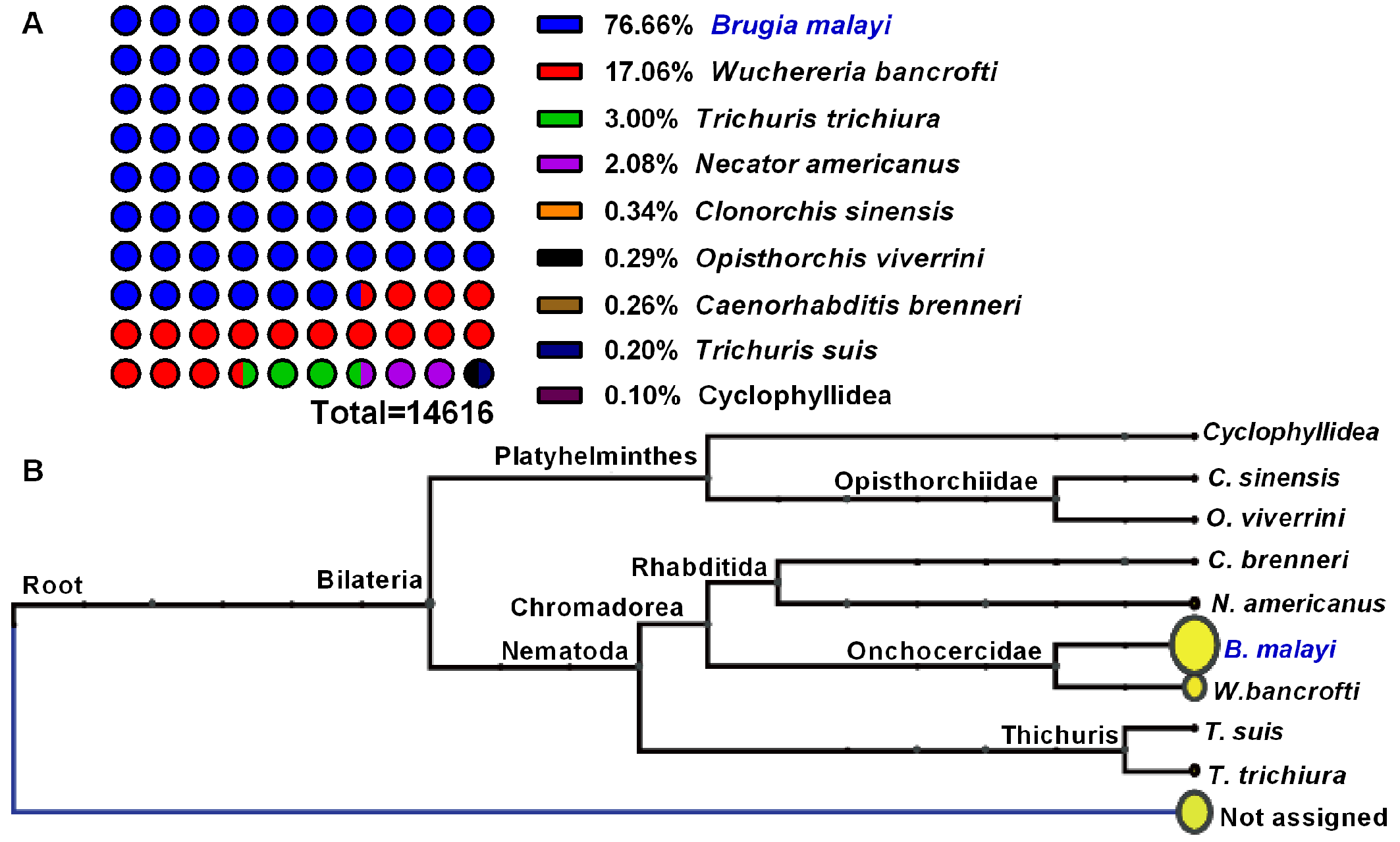


**Fig. S2** Typing of blast hits after analyzing the eye discharge sample and detail of the phylogenetic MEGAN output. **a** The ratio of potential parasites in non-human sample. **b** MEGAN output shows the phylogenetic relationship of species hitting eye discharge sample.
