# Supplementary material for: Diagnosis of a malayan filariasis case using a shotgun diagnostic metagenomics assay
Source: Parasit Vectors. 2016 Feb 16;9:86. doi: 10.1186/s13071-016-1363-2 (PMC4754835; doi:10.1186/s13071-016-1363-2)
Supplement: Additional file 4: Figure S3. — Typing of blast hits after analyzing subcutaneous tissue sample and details of the phylogenetic MEGAN output. (DOC 198 kb) [file 13071_2016_1363_MOESM4_ESM.doc]

**
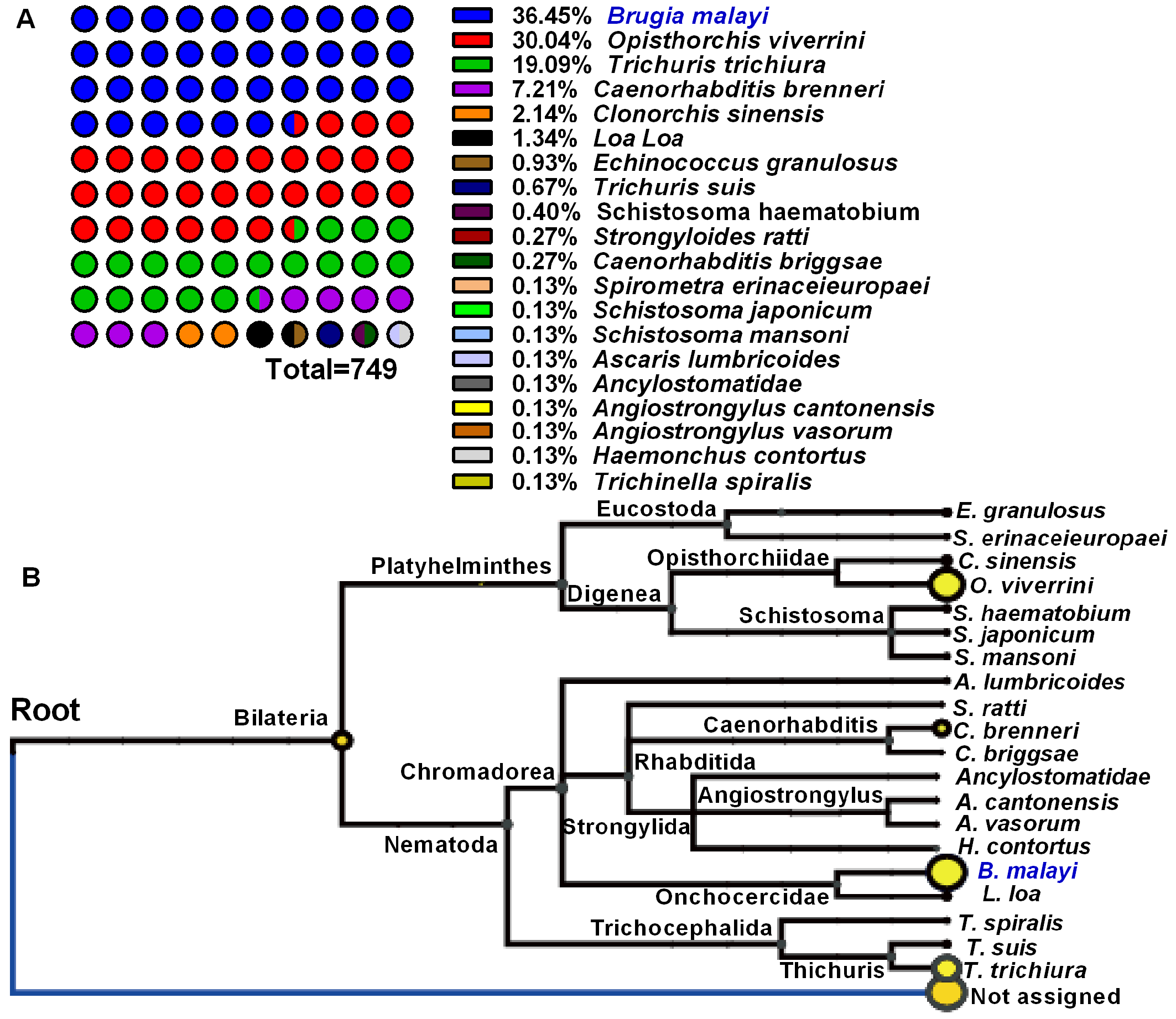
**

**Fig. S3** Typing of blast hits after analyzing subcutaneous tissue sample and detail of the phylogenetic MEGAN output. **a** The ratio of potential parasites in non-human sample. **b** MEGAN output shows the phylogenetic relationship of species hitting subcutaneous tissue sample.
